# Path Meander of Male Codling Moths (*Cydia pomonella*) Foraging for Sex Pheromone Plumes: Field Validation of a Novel Method for Quantifying Path Meander of Random Movers Developed Using Computer Simulations

**DOI:** 10.3390/insects11090549

**Published:** 2020-08-19

**Authors:** Christopher Adams, Jeffrey Schenker, Paul Weston, Lawrence Gut, James Miller

**Affiliations:** 1 Department of Entomology, Michigan State University, East Lansing, MI 48824, USA; gut@msu.edu (L.G.); Miller20@msu.edu (J.M.); 2 Department of Horticulture, Oregon State University, Hood River, OR 97031, USA; 3 Department of Mathematics, Michigan State University, East Lansing, MI 48824, USA; schenke6@msu.edu; 4 Graham Centre for Agricultural Innovation, Charles Sturt University, Wagga Wagga, New South Wales 2678, Australia; pweston@csu.edu.au

**Keywords:** random walkers, optimal foraging, resource finding, mover simulations

## Abstract

**Simple Summary:**

Measures of insect movement patterns are key to understanding how insects forage for resources and mating opportunities in their environment. Directly observing large numbers of these small organisms can be extremely challenging, especially for flying insects in low light conditions such as codling moth (*Cydia pomonella*), a key pest of apple. Here we provide a novel approach to indirectly measure the path meander of randomly moving organisms. Computer simulations were used to simulate insect movement across a wide range of possible movement patterns, measured in circular standard deviation (c.s.d.) of turn angles between track segments. For each c.s.d., the pattern of catch across a rectangular grid of traps was plotted and the resulting exponential decay constant (k) of the fitted lines were used to generate a standard curve describing this linear relationship. Using this standard curve, field data from target organisms caught in the described trapping grid can reveal the pattern of movement employed by these movers. Here we have demonstrated methodology for indirect measure of the movement patterns employed by random walkers such as *C. pomonella*. While we employed codling moth as our model system, we suggest this approach could prove useful in a wide range of other systems.

**Abstract:**

Measures of path meander are highly relevant to studies of optimal foraging by animals. However, directly recording paths of small animals such as insects can be difficult because of small size or crepuscular activity. Computer simulations of correlated random walkers demonstrated that the rates of decay in captures across a rectangular grid of traps when movers were released at its corner can be used to produce calibration curves for quantifying path meander indirectly. Simulations using spatial parameters matching those previously documented for male codling moths (*Cydia pomonella* (L.)) foraging for female pheromone plumes in the field predicted that meander, as measured in circular standard deviation (c.s.d.) of turn angles between track segments, should be ca. 50° and 30° when the target population density is high vs. low, respectively. Thus, if optimized, the mean value measured for *C. pomonella* populations encountering an unknown target density should fall between these limits. We recorded decay in *C. pomonella* catch across a 5 × 5 grid of pheromone-baited traps each separated by 15 m on 39 occasions where batches of ca. 800 males were released 10 m outside the corner of trapping grids arranged in five large Michigan apple orchards. This decay constant was translated into mean c.s.d value for path meander using the standard curve generated by the computer simulations. The measured decay constant for *C. pomonella* males was negative 0.99 ± 0.02 (S.E.M.), which translates to a path meander of 37 ± 2° c.s.d. Thus, the measured path meander of 37° fell between the 50° and 30° values optimal for dense and sparse populations, respectively. In addition to providing a rare documented example of optimal foraging for odor plumes, this research offers proof-of-concept for a novel approach to quantifying path meander of movers that could prove useful across diverse taxa.

## 1. Introduction

A presumption of behavioral ecology is that evolution will refine animal locomotory behaviors such that the average path taken while foraging will maximize the probability of finding critical resources, while minimizing time, energy, and bodily risk [1,2,3,4,5,6,7]. But, actual proof of this precept is rare. Animals such as lions, hawks, and predatory fishes are thought to fulfill this expectation via saltatory patterns of movement involving both cruising the habitat for prey and ambushing [8], all the while relying heavily on learning and memory. On the other hand, simpler animals like microorganisms [9,10], nematodes [11,12], and insects [13,14,15,16,17,18,19] may rely only on random walks (flights) [20,21,22] to bring them into contact with sensory cues from a resource that then orient the last legs of a foraging excursion [18]. An intriguing feature of biological random walks is their time-averaged symmetry in the frequency and severity of left vs. right turns between locomotory steps such that the resulting frequency histogram becomes smoothly Gaussian, e.g., Figure 7a of [23], Figure 2 of [24], and Figure 2b of [17]. Moreover, the spread (circular standard deviation (c.s.d.)) of such symmetrical frequency distributions of turns can vary with animal species from very little to strong forward-bias [25,26]. Random walkers executing frequent severe turns (high path meander) are more likely to find nearby than distant resources, while those executing gradual turns (low path meander) are more likely to find distant than close resources. Some random walkers benefit by actively increasing their c.s.d. for turning (intensifying local “search”) upon detecting resource cues and then narrowing c.s.d. (intensifying distant “search”) when these cues are no longer detected [5,27,28,29,30]. Spread of turn angle distributions during foraging is a critical biological trait [5,18,31], shown to be heritable [32]. We suggest that diversifying approaches to its measurement is a worthy research endeavor.

A readily accessible and direct approach to quantifying relative path meander of slow-moving foragers is to measure, and then plot, a frequency histogram for turns between uniform segments of an animal’s foraging track [33] recorded by, e.g., videography [34,35]. A suitable unit of path length for a walker is one body length [18], while that for a flyer may be a meter or more [36,37]. Upon slow-motion video play-back, the position of track segment ends can be recorded by digitizing software and then the angle struck by the preceding vs. current segment of track can be measured or computed, using computer software. Measurements from at least 100 steps are usually required to generate a smooth frequency histogram whose symmetry can be tested statistically. One c.s.d. is computed as the square root of the variance. For Gaussian outcomes, one c.s.d. to the left and right of the mean turn angle (straight ahead for classical random walkers) will encompass 68% of the data.

Foraging tracks for fast and far-ranging animals can be approximated from data sets recorded by e.g., acoustical [38] or radio [39,40,41,42,43,44] telemetry, where the mover carries a transmitter whose remotely detected signal is positioned geographically by triangulation. Increasingly, the transmitters carried by large animals are designed for detection by Earth-orbiting satellites and GPS positioning [38,43]. Although this approach works well for quantifying large-scale and migratory movements, it is suitable for quantifying randomness of local walks only when the frequency of positioning is extraordinarily high.

Our research into the flight behaviors of the male codling moth (*Cydia pomonella* (L.)), the key pest of apple and pear crops, foraging for female sex pheromone plumes presented technical challenges to a direct tracking approach. Not only can this 1-cm-long moth not carry a suitable transmitter or reflector, but it also flies under very low light levels, close to its host apple trees at speeds of ca. 1 m/s, making nocturnal video recordings prohibitive. We wondered whether our goal of measuring male codling moth’s flight meander, before encounter with a pheromone plume, might be accomplished by an indirect approach of analyzing patterns of captures in an array of pheromone-baited traps rather than by direct tracking.

## 2. Insights from Computer Simulations

### Methods and Results

**Figure 1:**
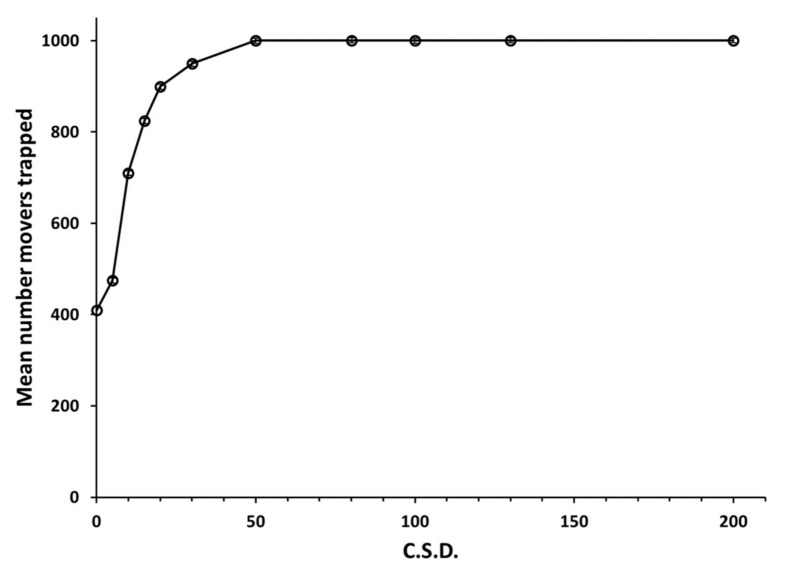
Mean capture of populations of 1000 random walkers released near the center of a 7 × 7 grid of 2 × 2 unit traps evenly spaced 20 units apart, as a function of circular standard deviation (c.s.d.). All movers were captured at c.s.d. 50° and above. S.E.M. bars around means are so tiny as to be barely visible.

The proprietary MultiMover software used here for random-walk simulations was written in the late 1990s by author P. A. Weston and previously featured in much of the work reported in Miller et al. (2015). Copies of this flexible and user-friendly program, written for the PC and previously distributed on a limited basis for teaching purposes, may be obtained upon reasonable request by contacting pweston@csu.edu.au. Its features and functions parallel what can be achieved using MATLAB^®^, but with superior visualizations conducive to behavioral studies. Here, we first quantified how changes in c.s.d. influenced escape of random walker populations (1000 movers for each of 12 runs) released near the center of a 7 × 7 grid of 2 × 2 unit traps evenly spaced 20 units apart. A small trap was used to approximate the small pheromone plume measured for codling moth traps [19]. Each mover initially took a step of 1 unit (1/1000th the width of the computer monitor) following an independently and randomly assigned heading. Thereafter, headings for each of 2000 steps by each mover were independently and randomly picked by the computer’s random number generator from a Gaussian distribution of possible headings centered on straight ahead and having a specified c.s.d. for each mover population. Movers encountering any trap were held for the duration of the run and automatically counted. Those not contacting traps were free to range among the traps and beyond the grid in unbounded cyberspace. As expected and confirmed by Figure 1, movers with a large c.s.d. (high meander) quickly contact nearby traps before they express much movement away from their release point, whereas movers with a small c.s.d. (low meander) quickly escaped and traveled far outside the grid of traps, and seldom returned. These findings suggested that trapping experiments aimed at elucidating the c.s.d. of a population of movers would require an experimental design that allows large c.s.d. movers to fully express their movement patterns before capture, while increasing the probability of capture for small c.s.d movers.

**Figure 2:**
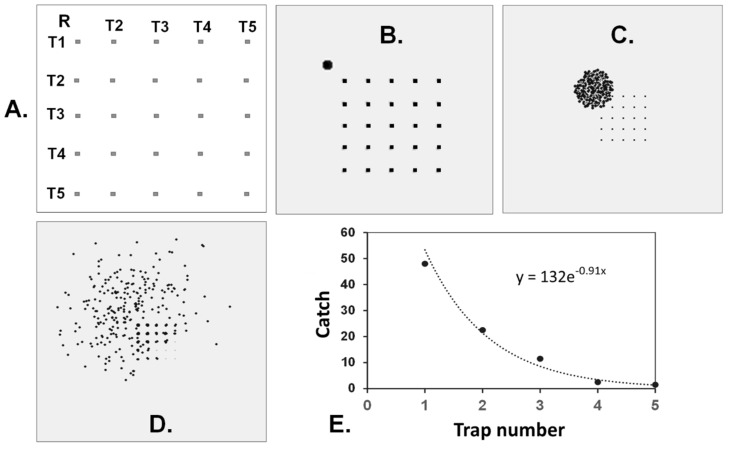
(**A**) Layout for grid of twenty-five 2 × 2-unit traps spaced 15 units apart (small rectangles) and the release point (R) of movers. Edge traps, whose mean catch numbers were used for graphing data to quantify a decay constant, are numbered by relative distance away from the release point. (**B**–**D**) show final destinations of movers after 5, 100, and 400 steps. The spatial scale for panels varies. Movers caught on traps in (**D**) enlarge trap appearance. (**E**) Plot of mean catch by trap number (as in A) after 400 movers released at R, took 400 steps of 1 unit with a circular standard deviation (c.s.d.) of 30°. The mean of paired traps was plotted for traps designated T2–T5.

As preliminarily explored in [18] (Chapter 7), we therefore shifted the cyber exploration to releasing the mover populations just outside a corner of a rectangular grid of traps and measuring captures by individual traps within it. Comprehensive exploration of catch data revealed that changes in c.s.d could be detected by graphing catch data of edge traps, numbered by their relative distance away from the release point. Interior traps, not included in the analysis, prevented the majority of movers from returning to the edge traps, after their initial pass. Figure 2A documents the spatial arrangement and trap-numbering system of a cyber experiment having conditions selected to approximate those of a planned field experiment using actual codling moths. Figure 2B–D reveals the positions of individual movers after 5, 100, and 400 steps by a population of 400 cyber random walkers operating with a c.s.d. of 30° as they progressively dispersed, including into and through a grid of 25 two x two unit traps spaced at 15 units. Because the preponderance of catch occurred in the edge traps, labeled T1 through T5 in Figure 2A, only these catch data were used to quantify how catch shifted with distance from the release point. As per Figure 2E, catch declined smoothly and exponentially with distance so as to yield a decay constant (k) of negative 0.91.

**Figure 3:**
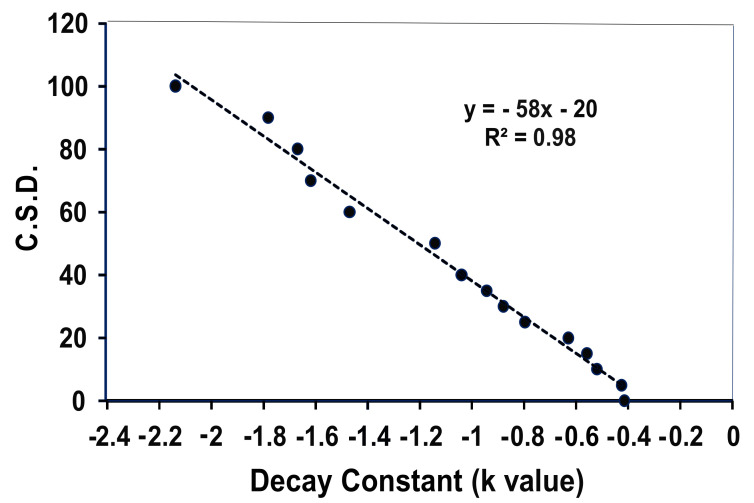
Standard curve inter-relating decay constant (k) with circular standard deviation (c.s.d.). This graph was generated using the computer-simulation procedures of Figure 2 and a run time of 400 steps. Ten replicate runs generated 10 k values for each c.s.d. S.E.M. values around the mean e-values were too small to be visible.

The next step was quantifying decay constant of catch with distance from the release point as a function of c.s.d. magnitude for cyber random walkers. That relationship conveniently turned out to be linear (Figure 3) so as to yield a useful standard curve for reading out c.s.d. values from measured values for decay constant in catch of movers released in the configuration of Figure 2A.

**Figure 4:**
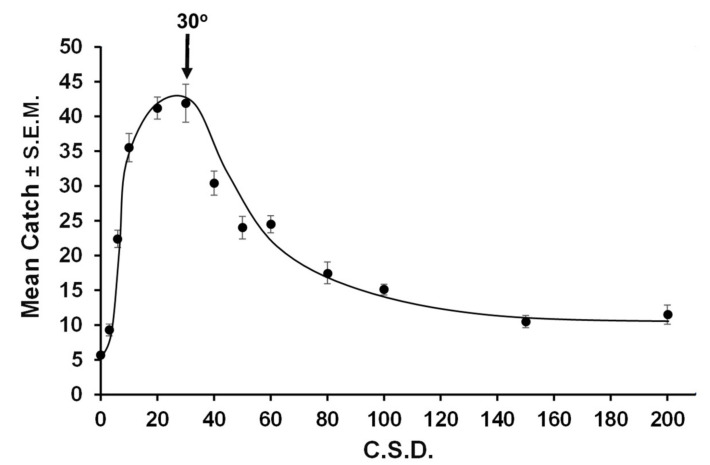
Catch as a function of circular standard deviation (c.s.d.) by populations of 5000 movers randomly seeded within a starting arena of 1000 × 600 units of an unbounded cyber arena, where they took 3000 steps with one 2 × 2 units trap at its center.

Finally, cyber experiments with random walkers clarified what c.s.d. values might maximize contacts with targets under the conditions experienced by codling moths [19]. As already noted in Figure 1, finding of a small resource presented at high density (1 per 300 square computer units) became maximal at a c.s.d. of 50°. A 2000-fold decrease in target density (1 per 600,000 square cyber units) was achieved by randomly seeding 5000 random walkers into a 1000 × 600 unbounded cyber arena having a single 2 × 2 unit trap at its center and recording catch after 3000 steps under varying c.s.d. values. At this low target density, the optimal c.s.d. sharpened and decreased to ca. 30° (Figure 4). Movers with a larger c.s.d. performed poorly because appreciable dispersion was required for most movers to reach the target. When the cyber arena was enlarged beyond 1000 × 600 cyber units, the run times were extended beyond 3000 steps, and the target size was increased, the optimal c.s.d. trended lower than 30° and peak catch flattened (J. H. S., unpublished data). However, these conditions do not match those that codling moths would be likely to encounter in the field [19,45].

This background knowledge set the stage for interpreting experimental results trapping codling moths in the field under a comparable spatial setup to that of Figure 2A. We postulated that males would exhibit a mean c.s.d. falling between 30° and 50°, because such an intermediate value would perform reasonably well for either a dense or sparse female population, both of which can be encountered by natural codling moth populations across generations [45].

## 3. Differential Catch of Codling Moth Males in a Trapping Grid in Apple Orchards

### 3.1. Methods

To put this approach to measuring path meander to its first test with real animals, we released batches of ca. 800 laboratory-reared codling moths marked with fluorescent powders of unique color 10 m outside of the corner of a 5 × 5 grid of optimized traps separated by 15 m. Experiments were conducted in orchards located in Sparta Michigan planted with Gala and Honey Crisp trees trained to trellis wire, ca. 3.5 m in height and spaced at 2.5 m. Trapping grid consisted of 25 identical orange delta traps (Pherocon VI; Trécé Inc., Adair, OK, USA) hung in the top third of the canopy and equipped with a CML2 gray septum lure (Trécé Inc.) held aloft of the sticky liner by a pin through the roof. Thirty-nine replicates of this experiment were accumulated between 6 June and 9 July of 2015. In some cases, differently marked batches of moths were released at all four corners of a trapping array. Captures in all traps were recorded daily, and catch for each moth batch was plotted as in Figure 2 and c.s.d. computed using the standard curve of Figure 3.

### 3.2. Results and Discussion

**Figure 5:**
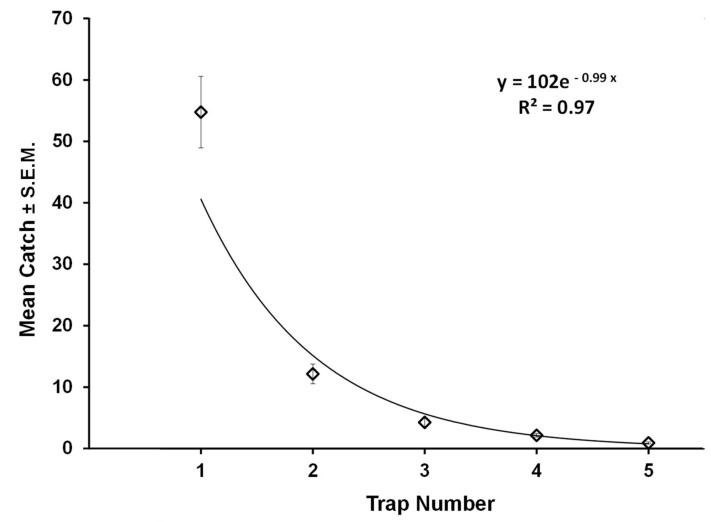
Decay curve averaged across all 39 replicates of codling moth males penetrating a 5 × 5 grid of traps in the field. An exponent of negative 0.99 equates to a circular standard deviation (c.s.d.) of 37°.

**Figure 6:**
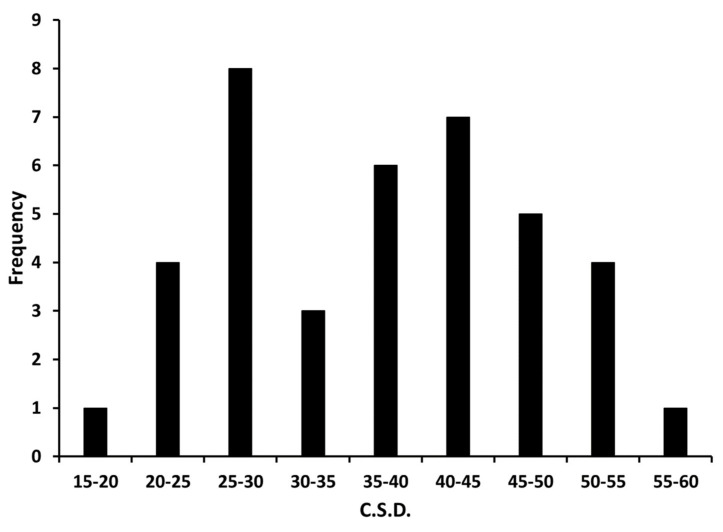
Frequency histogram of the 39 individual determinations of circular standard deviation (c.s.d.) for codling moth foraging for pheromone plumes in a Michigan apple orchard.

Average decline in catch of male codling moths as a function of trap position in the grid (numbered as per Figure 2) is shown in Figure 5. The mean decay constant (*k* value) arising from the 39 individual determinations was −0.99 ± 0.02 (S.E.M.), which interpreted using the standard curve of Figure 3, equates to a c.s.d. of 37 ± 2° (S.E.M.). Indeed, this value falls between 30° and 50°, as postulated for an optimal forager under these given conditions. The frequency histogram of the 39 measured meander values (Figure 6) shows appreciable variability around the mean of 37° c.s.d., some of which could be measurement error. However, it is notable that the range in measured average meander values for *C. pomonella* males never fell below 15° or above 60°, which would indicate considerable variation in foraging behavior rather than conversion around a single strategy, as one might expect. Not revealed in these data from our experiment exploiting the law of large numbers, is the amount of individual variation that might occur in this codling moth population. One can speculate that our populations of released males contained some individuals with an ethotype better suited to finding sparse females (very low meander) while others with high meander were preadapted for best efficiency under a dense female population. But, the current data do not provide sufficient power to test that possibility with confidence.

## 4. Conclusions

The mechanisms male insects use to efficiently navigate to the source of a detected sex pheromone plume have been very well documented both in the laboratory and field [36,46,47,48]. The current study is one of the first cases actually providing empirical evidence for optimal foraging by an animal in the ranging stage before first contact with a pheromone. Moreover, the excellent pattern match of codling moth field data (Figure 5) to that generated by computer-simulated random walkers (Figure 2E) operating under similar spatial conditions, provides further confirmation that animals like small moths do forage by correlated random walks. Further, the agreement between computer simulations and field data is evidence that, for this species, two dimensional modeling can be translated into three dimensional insect movement, and we invite others to utilize this powerful tool. Finally, this research breaks new ground in demonstrating how a trapping grid can be used to quantify path meander by random walkers. While we employed codling moths as our model system, we suggest this approach could prove useful in a wide range of other biological, and perhaps non-biological systems. In particular, predicting the spread of invasive species is intrinsically tied to movement patterns and path meander. In addition, we see applications in fields such as swarm robotics for environmental monitoring systems [49] where understanding and programing optimal foraging meander directly parallels this work. While calibrations will be required for each situation, we have demonstrated that movement patterns of populations of movers, too small for direct tracking, can be indirectly measured. Computer simulations can be used to produce standard curves for interrelating c.s.d. for turning distributions and decay constant in catch across a given gird of traps, and properly calibrated, can measure c.s.d. in real world systems.

## References

[1] Katz P.L. (1974). A long-term approach to foraging optimization. Am. Nat..

[2] Pyke G.H. (1978). Are animals efficient harvesters?. Anim. Behav..

[3] Stephens D.W., Krebs J.R. (1986). Foraging Theory.

[4] Parker G.A., Smith J.M. (1990). Optimality theory in evolutionary biology. Nature.

[5] Bell W.J. (1991). Searching Behaviour: The Behavioural Ecology of Finding Resources.

[6] Viswanathan G.M., Buldyrev S.V., Havlin S.M., da Luz G.E., Raposo E.P., Stanley H.E. (1999). Optimizing the success of random searches. Nature.

[7] Sakiyama T., Gunji Y.-P. (2013). Emergence of an optimal search strategy from a simple random walk. J. R. Soc. Interface.

[8] O’Brien W.J., Browman H.I., Evans B.I. (1990). Search strategies of foraging animals. Am. Sci..

[9] Hill N.A., Hädler D.P. (1997). A biased random walk model for the trajectories of swimming micro-organisms. J. Theor. Biol..

[10] Hall R.L. (2015). Amoeboid movement as a correlated random walk. J. Mathmat. Biol..

[11] Broadbent S.R., Kendall D.G. (1953). The random walk of *Trichostrongylus retortaeformis*. Biometrics.

[12] Helms S.J., Avery L., Stephens G.J., Shimizu T.S. (2015). Modelling the ballistic-to-diffusive transition in nematode motility reveals low-dimensional behavioral variation across species. arXiv.

[13] Kareiva P.M., Shigesada N. (1983). Analyzing insect movement as a correlated random walk. Oecologia.

[14] Crist T.O., MacMahon J.A. (1991). Individual foraging components of harvester ants: Movement patterns and seed patch fidelity. Insectes Sociaux.

[15] Nagasaka K., Mase K., Okada E., Yamamoto T. (1998). Analyzing dispersal of silkworm larvae as a random process. J. Sericultural Sci. Jpn..

[16] Byers J.A. (2001). Correlated random walk equations of animal dispersal resolved by simulations. Ecology.

[17] Bengtsson G., Nilsson E., Ryden T., Wiktorsson M. (2004). Irregular walks and loops combine in small-scale movement of a soil insect: Implications for dispersal biology. J. Theor. Biol..

[18] Miller J.R., Adams C.G., Weston P.A., Schenker J.H. (2015). Trapping of Small Organisms Moving Randomly: Principles and Applications to Pest Monitoring and Management.

[19] Adams C.G., Schenker J.H., McGhee P.S., Gut L.J., Brunner J.F., Miller J.R. (2017). Maximizing information yield from pheromone-baited monitoring traps: Estimating plume reach, trapping radius, and absolute density of *Cydia pomonella* (Lepidoptera: Tortricidae) in Michigan apple. J. Econ. Entomol..

[20] Berg H.C. (1993). Random Walks in Biology.

[21] Codling E.A., Plank M.J., Benhamou S. (2008). Random walk models in biology. J. R. Soc. Interface.

[22] Svensson G.P., Valeur P.G., Reynolds D.R., Smith A.D., Riley J.R., Baker T.C., Guy M., Poppy G.M., Löfstedt C. (2001). Mating disruption in *Agrotis segetum* monitored by harmonic radar. Entomol. Expt. Appl..

[23] Wehner R., Wehner S. (1990). Insect navigation: Use of maps or Ariadne’s thread?. Ethol. Ecol. Evol..

[24] Hill S., Burrows M.T., Hughes R.N. (2000). Increased turning per unit distance as an area-restricted search mechanism in a pause-travel predator, juvenile plaice, foraging for buried bivalves. J. Fish Biol..

[25] Lynch J.F., Balinsky E.C., Vail S.G. (1980). Foraging patterns in three sympatric forest ant species, *Prenolepis impairs*, *Paratrechina* and *Aphaenogaster rudis* (Hymenoptera: Formicidae). Ecol. Entomol..

[26] Pearce-Duvet J.M., Elamans C.P.H., Fenner D.F. (2011). Walking the line: Search behavior and foraging success in ant species. Behav. Ecol..

[27] Bond A. (1980). Optimal foraging in a uniform habitat: The search mechanism of the green lacewing. Anim. Behav..

[28] Nakamuta K. (1985). Mechanism of the switchover from extensive to area-concentrated search behavior of the ladybird beetle, *coccinella septempunctata bruckii*. J. Insect Phys..

[29] Nurzaman S.G., Matsumoto Y., Nakamura Y., Shirai K., Koizumi S., Ishiguro H. (2011). From Levy to Brownian: A computational model based on biological fluctuation. PLoS ONE.

[30] Ferran A., Ettifouri M., Clement P., Bell W.J. (1994). Sources of variability in the transition from extensive to intensive search in coccinellid predators (Homoptera: Coccinellidae). J. Insect Behav..

[31] Bovet P., Benhamou S. (1991). Optimal sinuosity in central place foraging movements. Anim. Behav..

[32] Sokolowski M. (1980). Foraging strategies of *Drosophila melanogaster*: A chromosomal analysis. Behav. Genet..

[33] Edelhoff H., Signer J., Balkenhol N. (2016). Path segmentation for beginners: An overview of current methods for detecting changes in animal movement patterns. Mov. Ecol..

[34] Riley J.R., Wratten S.D. (1993). Flying Insects in the field. Video Techniques in Animal Ecology and Behaviour.

[35] Noldus L.P., Spink A.J., Tegelenbosch R.A.J. (2002). Computerized video tracking, movement analysis and behaviour recognition in insects. Comput. Electron. Agric..

[36] Cardé R.T., Cardé A.M., Girling R.D. (2012). Observations on the flight paths of the day-flying moth *Virbia lamae* during periods of mate location: Do males have a strategy for contacting the pheromone plume?. J. Anim. Ecol..

[37] Bau J., Cardé R.T. (2015). Modeling optimal strategies for finding a resource-linked windborne odor plume: Theories, robotics and bionomic lessons from flying insects. Integr. Comp. Biol..

[38] Espinoza M., Farrugia T.J., Webber D.M., Smith F., Lowe C.G. (2011). Testing a new acoustic telemetry technique to quantify long-term, fine-scale movements in aquatic animals. Fish. Res..

[39] Jain V.R., Bagree R., Kumar A., Ranjan P. (2008). wildCENSE: GPS Based Animal Tracking System. Proceedings of the 2008 International Conference on Intelligent Sensors, Sensor Networks and Information Processing.

[40] Riecken U., Ulrike R. (1996). Use of radio telemetry for studying dispersal and habitat use of *Carabus coriaceus* L.. Annl. Zoo. Fenn..

[41] Cagnacci F., Boitani L., Powel R.A., Boyce M.S. (2010). Animal ecology meets GPS-based radiotelemetry: A perfect storm of opportunities and challenges. Philos. Trans. R. Soc. B.

[42] Vinatier F., Chailleux A., Duyck P., Salmon F., Lescourret R.P., Tixier P. (2010). Radiotelemetry unravels movements of a walking insect species in heterogeneous environments. Anim. Behav..

[43] Wikelski M., Moxley J., Eaton-Mordas A., Lopez-Uribe M.M., Holland R., Moskowitz D., Roubik D.W., Kays R. (2010). Large-range movements of neotropical orchid bees observed by radio telemetry. PLoS ONE.

[44] Walden-Schreiner C., Leung Y.-F., Kuhn T., Newburger T. (2018). Integrated direct observation and GPS tracking to monitor animal behavior and resource management. Environ. Monit. Assess..

[45] Trematerra P., Gentile P., Sciarretta A. (2004). Spatial analysis of pheromone trap catches of codling moth (*Cydia pomonella*) in two heterogeneous agroecosystems, using geostatistical techniques. Entomology.

[46] Witzgall P., Stelinski L., Gut L., Thomson D. (2008). Codling moth management and chemical ecology. Annu. Rev. Entomol..

[47] Thomson D.R., Gut L.J., Jenkins J.W., Hall F.R., Menn J.J. (1999). Pheromones for insect control: Strategies and successes. Biopesticides Use and Delivery.

[48] Allison J.D., Cardé R.T. (2016). Pheromone Communication in Moths.

[49] Tiwari K., Chong N.Y. (2019). Multi-Robot Exploration for Environmental Monitoring.

